# Bone-Targeted Agents for the Management of Breast Cancer Patients with Bone Metastases

**DOI:** 10.3390/jcm2030067

**Published:** 2013-08-19

**Authors:** Demetrios Simos, Christina L. Addison, Iryna Kuchuk, Brian Hutton, Sasha Mazzarello, Mark Clemons

**Affiliations:** 1 Division of Medical Oncology, Department of Medicine, The Ottawa Hospital Cancer Centre, University of Ottawa, Ottawa Hospital Research Institute, Box 900, 501 Smyth Road, Ottawa, Ontario K1H8L6, Canada; E-Mails: dsimos@toh.on.ca (D.S.); ikuchuk@toh.on.ca (I.K.); smazz052@uottawa.ca (S.M.); 2 Centre for Cancer Therapeutics, The Ottawa Hospital Research Institute, Departments of Medicine & Biochemistry Microbiology & Immunology, University of Ottawa, Box 926, 501 Smyth Road, Ottawa, Ontario K1H8L6, Canada; E-Mail: caddison@ohri.ca; 3 Centre for Practice Changing Research & Ottawa Hospital Research Institute, Box 201, 501 Smyth Road, Ottawa, Ontario K1H 8L6, Canada; E-Mail: bhutton@ohri.ca

**Keywords:** bone metastases, bone-targeted therapy, skeletal-related events, bisphosphonates, denosumab

## Abstract

Despite advances in adjuvant therapy for breast cancer, bone remains the most common site of recurrence. The goal of therapy for these patients is palliative and focused on maximizing the duration and quality of their life, while concurrently minimizing any disease or treatment-related complications. Bone metastases predispose patients to reduced survival, pain, impaired quality of life and the development of skeletal-related events. With an increased understanding of the pathophysiology of bone metastasis, effective treatments for their management have evolved and are now in widespread clinical use. This article will discuss the pathogenesis of bone metastases and review the key clinical evidence for the efficacy and safety of currently available systemic bone-targeted therapies in breast cancer patients with an emphasis on bisphosphonates and the receptor activator of nuclear factor kappa B ligand (RANKL) inhibitors. We will also discuss novel strategies and therapies currently in development.

## 1. Introduction

Bone remains the most common site of breast cancer recurrence [1,2,3]. The development of bone metastases is associated with reduced survival, impaired quality of life and pain. In addition, bone metastases can undermine the structural integrity of the skeleton and predispose patients to skeletal complications, collectively known as skeletal-related events (SREs). SREs typically include: Radiotherapy or surgery to the bone, pathologic fractures, spinal cord compression and hypercalcemia of malignancy [2,4,5,6]. SREs are associated with significant morbidity and mortality, and their avoidance is an essential component of patient management [7,8,9,10,11].

Over the past two decades, bone-targeted agents, such as bisphosphonates and the receptor activator of nuclear kappa ligand (RANKL) inhibitor, denosumab, have emerged as effective options for the treatment of women with breast cancer that has metastasized to the bone [12,13,14]. Their use in combination with anti-cancer therapies (*i.e.*, chemotherapy and hormonal therapy) is supported by clinical trial evidence showing efficacy for reducing the number of SREs and delaying the onset of SREs [15,16]. As a result, these agents are now considered the standard of care for the treatment of breast cancer patients with bone metastases [17].

The following review will summarize the major clinical trial evidence for both the bisphosphonates and denosumab for the treatment of bone metastases from breast cancer. We will briefly discuss their proposed mechanisms of action as they relate to the pathophysiology of bone metastases. We will not discuss adjuvant bone-targeted therapies, as this is beyond the scope of this manuscript [18,19]. Finally, we will review some novel treatment strategies and newer therapies currently under investigation that may be entering clinical practice in the future.

## 2. Pathogenesis of Breast Cancer Bone Metastases

Normal bone is continuously renewed by remodeling, a tightly controlled process that involves a balance between osteoclast-mediated bone resorption and osteoblast-mediated bone formation [5]. Disturbance in this balance will either lead to net bone loss or formation. Breast cancer cells that metastasize to the bone marrow do not directly cause bone destruction on their own [17,20]. Instead, they alter the bone microenvironment, leading to an uncoupling of the balance between bone resorption and formation [5,17,20,21]. This process is complex, but in essence, the presence of breast cancer cells in the bone ultimately leads to excessive bone resorption via upregulation of osteoclasts leading to characteristic lytic metastases and tumor propagation [5,17,20,21,22]. The disruption of normal bone remodeling by metastatic breast cancer cells ultimately sets up a “vicious cycle” that propagates the process of bone destruction [5,10].

## 3. Mechanism of Action of Bone-Targeted Agents

### 3.1. The Bisphosphonates

Bisphosphonates are stable drug analogues of naturally occurring inorganic pyrophosphate that can: (i) Inhibit processes essential for osteoclast survival and (ii) Promote osteoclast apoptosis [23]. As a result of their anti-osteoclastic effects, bisphosphonates have the ability to modulate bone turn over and diminish tumour-mediated bone resorption and destruction [23]. There are two main classes of bisphosphonates; the amino- and the non-amino bisphosphonates [23,24]. Given their greater potency, the amino-bisphosphonates are generally used clinically [23,24]. There are currently four bisphosphonates available for use to treat women with breast cancer bone metastases; clodronate, pamidronate, ibandronate and zoledronic acid. All except clodronate are amino-containing bisphosphonates [23].

### 3.2. Denosumab

Denosumab is a fully human monoclonal antibody that binds to RANKL and prevents it from interacting and activating its receptor, RANK [25,26]. RANK is found on the surface of osteoclasts and osteoclast precursors, and pre-clinical models identified the RANKL-RANK interaction as a key mediator of osteoclastic activation [25,26]. By binding to RANKL, denosumab decreases cancer-induced bone destruction by suppressing osteoclast-mediated bone resorption.

## 4. Clinical Trial Evidence for Bone-Targeted Agents Used in the Management of Bone Metastases from Breast Cancer

### 4.1. The Bisphosphonates

#### 4.1.1. Clodronate

Clodronate was the first bisphosphonate widely studied in women with breast cancer metastatic to bone [27,28]. Subsequent larger placebo-controlled trials confirmed that daily oral clodronate was effective for reducing the incidence of SREs [29,30,31] (Table 1).

**Table 1:** Placebo controlled trials of bisphosphonates available for clinical use.

RCT first author, year, sample size, comparison made	Results	*p*-Value
**Clodronate**		
Patterson *et al*., 1993 [29] *N* = 173 Clodronate 1600 mg orally daily *vs*. placebo	1. Hypercalcemia	
	No. of patients developing hypercalcemia: 20 *vs*. 31	>0.05
	↓ Episodes of terminal hypercalcemia: 7 *vs*. 17	0.05
	↓ Total hypercalcemic events (events per 100 patient years): 28 *vs*. 52	0.01
	2. Fractures	
	Non-vertebral fractures (events per 100 patient years): 32 *vs.* 40	>0.05
	↓ Vertebral fractures (events per 100 patient years): 84 *vs.* 124	0.025
	↓ Rate of vertebral deformity (events per 100 patient years): 252 *vs.* 168	<0.001
	3. Requirement for radiotherapy to bone	
	No. of patients requiring radiotherapy: 34 *vs.* 42	>0.05
	No. of courses of radiotherapy (events per 100 patient years): 75 *vs.* 89	>0.05
	4. Other	
	Overall skeletal morbidity (events per 100 patient years): 219 *vs.* 305	<0.001
	No difference in survival between groups	
Kristensen *et al*., 1999 [30] *N* = 100 Clodronate 1600 mg orally daily *vs.* placebo	↑ Time to first skeletal related event	<0.015
	↓ Occurrence of fractures	<0.023
	No significant effect on quality of life Skeletal related effects are not sustained	
Tubiana-Hulin *et al*., 2001 [31] *N* = 144 Clodronate 1600 mg orally daily *vs.* placebo	↑ Time to new bone event: 244 *vs.* 180 days	0.05
	↓ Pain intensity (measured by visual pain scale)	0.01
	↓ Analgesic use	0.02
	
**Pamidronate**		
Conte *et al*., 1996 [32] *N*= 295 Pamidronate 45 mg i.v. every 3 weeks *vs.* placebo	↑ Time to disease progression: 249 *vs.* 168 days	0.02
	↑ Pain relief: 44% *vs.* 30% of patients	0.025
	
Hortobagyi *et al*., 1998 [33] *N* = 382 Pamidronate 90 mg i.v. every 3–4 weeks *vs.* placebo for 2 years	↓ Proportion of patients with any skeletal related complication at 15, 18, 21, and 24 months	<0.001
	↑ Median time to first skeletal related complication: 13.9 *vs.* 7.0 months	<0.001
	
Hultborn R. *et al*., 1999 [34] *N* = 404 Pamidronate 60 mg i.v. every 4 weeks *vs.* placebo	↑ Time to progression of pain	<0.01
	↑ Time to hypercalcemic events	<0.05
	↓ Skeletal related events	<0.01
	↑ Performance status scores	<0.05
	No change in pathologic fractures of long bones or pelvis No change in paralysis from vertebral compression No change in palliative radiotherapy or surgery to bone	
Theriault *et al*., 1999 [35] *N* = 372 Pamidronate 90 mg i.v. every 4 weeks *vs.* placebo for 2 years	↓ Skeletal morbidity rate at 12, 18, and 24 cycles	0.028, 0.023, 0.008
	↓ Skeletal complications at 24 cycles: 67% *vs.* 56%	0.027
	↑ Time to first skeletal complication: 10.4 *vs.* 6.9 months	0.049
	No difference in survival or objective response rate	
Lipton *et al*., 2000 [36] *N* = 754 Combined analysis and 24-month extension of Hortobagyi and Theriault trials above Pamidronate 90 mg i.v. every 3–4 weeks *vs.* placebo	↓ Skeletal morbidity rate: 2.4 *vs.* 3.7	<0.001
	↓ Total skeletal complications: 51% *vs.* 64%	<0.001
	↑ Median time to first skeletal complication: 12.7 *vs.* 7 months	<0.001
	No difference in median overall survival: 19.8 *vs.* 17.8 months	0.976
**Ibandronate**		
Body *et al*., 2003 [37] *N*= 466 Ibandronate 2 mg or 6 mg i.v. every 3–4 weeks *vs.* placebo for 2 years	Results for 6 mg ibandronate group:	
	↓ Skeletal morbidity period rate in patients receiving ibandronate 6 mg relative to placebo: 1.19 *vs.* 1.48 events	0.004
	↓ Mean number of bone events per patient: 2.65 *vs.* 3.64	0.032
	↑ Time to first skeletal related event: 50.6 *vs.* 33.1 weeks	0.018
	In general, the 6 mg dose of ibandronate fared better than the 2 mg dose	
Body *et al* ., 2004 [38] *N* = 564 Pooled analysis of 2 concurrent oral ibandronate trials Ibandronate 50 mg orally daily *vs.* placebo for 96 weeks	↓ Skeletal morbidity period rate: 0.95 *vs.* 1.18	0.004
	↓ Risk of skeletal related event: HR = 0.62	0.0001
	There was no significant ↑ in time to first skeletal related event or difference in the proportion of patients with an skeletal related event	
Heras *et al*., 2008 [39] *N* = 150 Ibandronate 6 mg i.v. every 4 weeks *vs.* placebo for 24 months	↓ Proportion of patients who experienced an skeletal related event: 36% *vs.* 48%	0.027
	↑ Time to first skeletal related event: 457 *vs.* 304 days	0.007
	↓ Risk of developing a skeletal related event by 32%: HR = 0.69	0.003
**Zoledronic acid**		
Kohno *et al*., 2005 [40] *N* = 228 Zoledronic acid 4 mg i.v. every 4 weeks *vs.* placebo for 1 year	↓ The rate of skeletal related events by 39%	0.027
	↓ Percentage of patients with at least one skeletal related event: 29.8% *vs.* 49.6%	0.003
	↑ Time to first skeletal related event: median not reached *vs.* 364 days	0.007
	↓ Risk of skeletal related events by 41%	0.019

RCT: Randomized controlled trial; No.: Number; *vs*.: *versus*; i.v.: Intravenous; HR: Hazard ratio.

#### 4.1.2. Pamidronate

The first large placebo-controlled randomized trial with pamidronate in women with breast cancer metastases to bone was performed over 20 years ago [31]. In this trial, pamidronate was administered orally on a daily basis instead of the usual intravenous dose on a 3–4 week basis. Despite having to reduce the dose in half from the initially planned 600 mg/day dose (due to significant nausea and vomiting), this study demonstrated that oral pamidronate significantly reduced hypercalcemia, bone pain, pathological fractures and the need for radiotherapy compared to the placebo group [29,31]. Moreover, this study highlighted that oral pamidronate can cause significant gastrointestinal toxicity and also led to further studies using the intravenous formulation.

Subsequent trials of pamidronate in patients with breast cancer assessed intravenous administration at three or four week intervals against placebo. The detailed findings from these trials are summarized in Table 1. The cumulative results from these trials showed that pamidronate significantly reduced the incidence of SREs and time to onset of SREs. This was independent of the underlying concurrent systemic chemotherapy or endocrine therapy. None of these trials showed either progression-free or overall survival advantage [32,33,34,35,36].

Contemporary practice is to use pamidronate at 90 mg intravenous every 3–4 weeks based on hypercalcemia studies [32,41,42]. The effect of pamidronate given intravenously at a lower dose *vs*. control on the incidence of SREs in women with metastatic breast cancer has also been investigated in two randomized trials (Table 1) [32,34]. The total dose given per unit time was similar in both trials, as one trial used a dose of 45 mg every three weeks and the other, a dose 60 mg every four weeks. Both trials demonstrated that pamidronate was significantly better than placebo at reducing the number of SREs and delaying the time to an SRE [32,34]. The findings from these studies are important clinically, as they provide the treating physician alternative dose options.

#### 4.1.3. Ibandronate

Ibandronate is a potent third-generation amino-bisphosphonate with oral and intravenous formulations. It is widely used in Europe and has demonstrated clinical efficacy on reducing and delaying SREs [37,38,39]. The recommended dosing is 50 mg orally per day or 6 mg intravenous every 3–4 weeks [37,38,39]. The results of the main ibandronate placebo-controlled clinical trials are summarized in Table 1.

#### 4.1.4. Zoledronic Acid

Zoledronic acid is a potent third-generation amino-bisphosphonate that can be administered intravenously over 15 min. Only one large study has investigated zoledronic acid compared to placebo [40]. This was a registration trial in Japan, where placebo was deemed an appropriate comparator arm as, at the time, no bisphosphonate had been approved for the treatment of metastatic bone disease. This study showed that zoledronic acid reduced SRE rates and delayed the time to the first SRE (Table 1) [40].

## 5. Head to Head Bisphosphonate Trials

The question of whether one bisphosphonate is more effective than others in controlling SREs in patients with breast cancer has been investigated for zoledronic acid relative to pamidronate [43] and ibandronate [44]. No head to head trial between clodronate and the other bisphosphonates in the metastatic setting have been completed. The main findings from these trials are summarized below and in Table 2.

**Table 2:** Comparative trials of bone-modifying therapies available for clinical use.

RCT first author, year, sample size, comparison made	Results	*p*-Value
**Zoledronic acid *vs.* Pamidronate**		
Rosen *et al*., 2003 [45] *N* = 1130 Zoledronic acid 4 mg or 8 mg i.v. *vs*. pamidronate 90 mg i.v. every 3–4 weeks for 12 months (8 mg group was dose-reduced to 4 mg, due to toxicity)	Results are for the 4 mg zoledronic acid group relative to pamidronate: Among all patients, the proportion of those who had a skeletal-related event was comparable: 43% *vs*. 45% In the subgroup of patients with lytic metastases (2° efficacy end-points), zoledronic acid:	
	↑ Median time to first skeletal-related event: 310 *vs*. 174 days	0.013
	↓ Mean annual incidence of skeletal events: 1.2 *vs*. 2.4 events per year	0.008
	↓ Risk of skeletal events by 30%: HR = 0.704	0.010
**Zoledronic acid *vs.* Ibandronate**		
Barrett-Lee *et al*., 2012 [44] *N* = 1405 Non-inferiority trial of Ibandronate 50 mg orally daily *vs*. zoledronic acid 4 mg i.v. every 3–4 weeks for 96 weeks	↓ Skeletal-related event rate for zoledronic acid relative to ibandronate: 0.444 *vs*. 0.543; however, this failed to meet the criteria for non-inferiority to zoledronic acid	0.017
	Similar time to first skeletal-related event: HR = 1.11	0.233
	No survival advantage	
**Denosumab *vs*. Zoledronic acid**		
Stopeck *et al*., 2010 [46] *N* = 2046 Denosumab 120 mg subcutaneously every four weeks *vs*. zoledronic acid 4 mg i.v. every four weeks	Denosumab:	
	↑ Time to first on-study skeletal-related events by 18%: HR = 0.82; median time to first skeletal-related event 24 months for zoledronic acid *vs*. not yet reached for denosumab	0.0001 (NI) 0.01 (S)
	↓ Risk of developing multiple skeletal-related events by 23%: Rate ratio: 0.77	0.001
	↓ Mean skeletal morbidity rate (ratio of the number of skeletal-related events per patient divided by the time at risk) by 22%: 0.45 *vs*. 0.58 events per patient per year	0.004
	No survival advantage	

RCT: Randomized controlled trial; No.: Number; *vs.*: *versus*; i.v.: intravenous; HR: Hazard ratio; NI: Non-inferiority; S: Superiority.

### 5.1. Pamidronate *vs*. Zoledronic Acid

Zoledronic acid was compared to pamidronate in 1130 patients with breast cancer and at least one metastatic bone lesion [43]. In this double blind, double dummy trial, patients were randomized to receive either pamidronate 90 mg intravenous or zoledronic acid 4 mg or 8 mg intravenous every 3–4 weeks for one year. Due to excess renal toxicity, the 8 mg dose of zoledronic acid was stopped, and patients in that arm received further treatment with 4 mg instead. The primary efficacy endpoint was the proportion of patients who experienced at least one SRE during the 13 months on the study. Overall, there was no difference between the zoledronic acid and pamidronate groups with respect to the primary endpoint. In the subgroup of patients with only lytic metastases, there was a statistically significant clinical benefit for zoledronic acid over pamidronate (prolonged time to first SRE and reduction in the annual incidence of skeletal events) (Table 2).

An extension of this trial to 25 months of follow-up included 412 patients with breast cancer [45]. There was no difference detected in the proportion of patients that experienced at least one SRE (46% in the zoledronic acid group and 49% in the pamidronate group). The skeletal morbidity rate (the ratio of the number of skeletal complications to the time on the trial) was reduced by 40% in the zoledronic acid group; however, this was not statistically significant (0.9 *vs*. 1.49 events per year). Multiple event analysis showed that zoledronic acid significantly reduced the risk of developing any skeletal complications by an additional 20% compared with pamidronate. The time to first SRE, skeletal morbidity rate and risk of skeletal complications were better controlled in the zoledronic acid group in patients receiving endocrine therapy, but not chemotherapy [45].

### 5.2. Ibandronate *vs*. Zoledronic Acid

The results of a large trial comparing oral ibandronate with zoledronic acid were recently reported in abstract form and are summarized in Table 2 [44]. The findings did not demonstrate that oral ibandronate was statistically inferior to zoledronic acid.

## 6. Meta-Analysis of Bisphosphonates and Their Effect on Skeletal-Related Events

The pooled analysis of data from eight studies that included 2189 breast cancer patients with bone metastases showed that bisphosphonates reduced the risk of developing an SRE by 17% compared to placebo (risk ratio: 0.83; 95% CI: 0.75–0.93; *p* = 0.001) [15]. There was no difference between oral or intravenous bisphosphonates (risk ratio: 0.84 *vs*. 0.83). The pooled data did not show a survival benefit in favor of bisphosphonates relative to placebo-control (risk ratio: 1.01; CI: 0.92–1.11) [15].

## 7. Denosumab

Two phase II trials of patients with bone metastases demonstrated that denosumab at doses ranging from 30 to 180 mg administered every four or 12 weeks was similar to intravenous bisphosphonates in suppressing bone turnover markers [47,48]. Despite the absence of evidence of differences among the majority of denosumab doses or between the four- and 12-week weekly administration on biomarker response, it was the 120 mg dose of denosumab every four weeks that has been evaluated in subsequent clinical trials.

### Denosumab *vs*. Zoledronic Acid

Denosumab (120 mg subcutaneously every four weeks) was compared head to head with zoledronic acid (4 mg intravenous every four weeks) in a pivotal large, double-blind, double-dummy phase III clinical trial involving 2046 women with metastatic breast cancer with bone metastases [46]. The efficacy endpoints were time to first SRE and time to first and subsequent SREs (multiple event analysis). This study showed that denosumab significantly delayed the time to first on-study SRE by 18%, reduced the risk of developing multiple SREs by 23% and delayed the median time to first onset SRE (26.4 months for zoledronic acid *vs*. not reached for denosumab). Denosumab was also shown to be superior to zoledronic acid in reducing markers of bone resorption after 13 weeks of follow-up. There was no progression-free or overall survival difference between the two arms [46] (Table 2).

## 8. Safety and Tolerability of Bisphosphonates

Bisphosphonates are generally well-tolerated; however, there are some common and potentially serious toxicities associated with their use. The most clinically significant toxicities are: Nephrotoxicity, hypocalcemia, acute phase reactions (fever, pain, fatigue) and osteonecrosis of the jaw (ONJ) [49,50,51,52,53]. These are all more commonly associated with intravenous bisphosphonates. Oral bisphosphonates may also cause significant gastrointestinal upset (nausea and vomiting, epigastric pain, esophagitis) [49,54].

### 8.1. Nephrotoxicity

Nephrotoxicity typically manifests as a rise in creatinine and occurs in 2%–8% of patients on intravenous bisphosphonates. Patients with underlying renal dysfunction are at greater risk [49]. The American Society of Clinical Oncology clinical practice guideline recommends careful clinical monitoring of renal function in all patients who are on active bisphosphonate treatment and, if renal impairment occurs, recommends discontinuation of the bisphosphonate until the serum creatinine has returned to within 10% of baseline [49]. Intravenous bisphosphonates are not recommended in patients with creatinine clearances less than 30 mL/min [49,50].

### 8.2. Hypocalcemia

Bisphosphonates cause a relatively rapid and prolonged drop in serum calcium and are the cornerstone of the modern day treatment of hypercalcemia; however, hypocalcemia may occur in up to 5% of patients on bisphosphonates [49]. Most of the trials that investigated their use in management of bone metastases supplemented the study participants with calcium and vitamin D (Table 1 and Table 2). It is generally recommended that patients on bisphosphonates be on concurrent calcium and vitamin D supplementation and have their serum calcium checked periodically [55,56].

### 8.3. Acute Phase Reactions

Fever, myalgias, bone pain and fatigue are quite common, occurring in up to one third of patients treated with intravenous bisphosphonates, and are most likely to occur following the first infusion [49,51]. They typically subside within 72 h and are believed to be a result of an acute flare of proinflammatory cytokines [51]. Given the likelihood of occurrence, patients about to start therapy with a bisphosphonate should be warned about the potential for developing such symptoms and reassured that they are transient and can be managed with supportive measures as required.

### 8.4. Osteonecrosis of the Jaw

Bisphosphonate-induced ONJ is perhaps the most dreaded potential complication of bisphosphonate therapy given its potential morbidity. It is defined as an area of exposed bone in the maxillofacial or mandibular area that has persisted for more than eight weeks, in a patient treated with bisphosphonates and no previous radiation therapy to the jaw [52,53,55]. Risk factors have been identified that may pre-dispose patients to ONJ, and these include: The use of high potency amino-bisphosphonates, prolonged bisphosphonate treatment, concurrent chemotherapy, underlying dental disease and a history of recent invasive dental procedures [53,57]. The incidence of ONJ in patients with breast cancer and bone metastases on a bisphosphonate ranges from 1.2%–2.4%, but can be as high as 10% in those who have multiple risk factors [49,53]. Patients should be screened for risk factors predisposing to ONJ, and any invasive dental procedures should be completed prior to initiation of therapy [53,58]. Patients should also be advised to let their dental care professional know that they are on bisphosphonate therapy. A careful examination of the oral cavity should be routinely performed, and prompt referral to a maxillofacial surgeon should be made for further management if ONJ is suspected.

### 8.5. Fragility Fractures

There is also increasing concern around the effects of long-term bone-targeted therapies on bone architecture with an increased risk of fragility fractures [59].

## 9. Safety and Tolerability of Denosumab

One of the proposed benefits of denosumab over bisphosphonates was the reduction in the requirement for renal monitoring [46,47,48]. That being said, however, widespread use of denosumab has only just begun, and its potential “real-world toxicities” (outside of the context of a clinical trial) are still evolving. At this point in time, the major concerning toxicities appear to be symptomatic hypocalcemia, renal toxicity and ONJ [14,16,46,49,60]. In studies, renal toxicity was reported as 4.9% in the denosumab group (as compared to 8.5% in the zoledronic acid group) [46]. Moreover, *ad hoc* analyses of a phase III trial that investigated denosumab in patients with bone metastases from prostate cancer, solid tumors and multiple myeloma, reported similar renal adverse events in both the denosumab and zoledronic acid groups (9.2% *vs*. 11.8%) [16]. Given these data, periodic monitoring of renal function and serum calcium should be considered in patients being treated with denosumab.

As with the potential for renal toxicity, the incidence of ONJ may also be higher for denosumab than previously thought. The pooled data from the three pivotal phase III trials of denosumab *vs*. zoledronic acid in metastatic bone disease showed that the incidence of ONJ was similar in both groups, at 1%–2% [60]. However, in the open label extension study of denosumab in patients with bone metastases from breast cancer, the incidence of ONJ of the jaw at five years was as high as 3.5%–4.7% [61].

## 10. Future Directions

Research focused on the identification of new therapeutic targets and on how to optimize or “personalize” the use of currently available bone-modifying therapies is ongoing [62]. The following section will address some novel agents and therapeutic strategies that are currently under investigation and, also, highlight some of the key questions looking to be answered with regards to optimizing or “personalizing” the use of currently available agents.

### 10.1. Novel Bone-Targeted Agents

Although use of denosumab or bisphosphonates has had a significant impact on the quality of life for patients with bone metastases, no prospective and appropriately powered study to date has demonstrated any improvement in either progression-free or overall survival. As such, new potential therapeutic options for bone metastatic patients are actively being investigated. The process of breast cancer mediated bone destruction is complex and involves many mediators, which ultimately lead to deregulation of the process of bone remodeling [63]. The factors mediating this process and how they interact with one another are slowly being elucidated and serve as potential new targets for novel therapies. Clinical trials evaluating novel drugs that affect both breast cancer and bone are ongoing and include many early phase trials [17,64]. The following section will briefly discuss some of these potential new therapies under clinical investigation.

### 10.2. Src Kinase Inhibitors

Src family kinases are known to play important roles in tumour cell proliferation and invasion, and as such, pharmacologic inhibitors to src family kinases have been developed. More recently, an important role for src has also been demonstrated in osteoclasts, whereby inhibition of src family members with selective tyrosine kinase inhibitors (TKIs) was shown to block osteoclast differentiation from precursor cells [65,66] and inhibit osteolytic tumor growth in preclinical models of bone metastasis [67]. Based on this rationale, phase II studies evaluating the effects of inhibition of src family members have been initiated in advanced breast cancer patients. Dasatinib monotherapy has shown some efficacy in advanced breast cancer patients [68,69,70]. A number of other clinical trials are ongoing with SRC inhibitors to evaluate bone turnover markers as a specified endpoint in addition to tumour responses in breast cancer, including randomized phase II studies with exemestane [71] or letrozole administered with or without dasatinib [72], a randomized phase II study of fulvestrant with or without dasatinib [73], a phase I/II study of dasatinib in combination with zoledronic acid [74] and a phase II study of dasatinib administered either once or twice daily in patients with breast cancer and bone metastases [75]. Saracatinib’s effects on bone markers have been evaluated in a randomized phase II trial *versus* zoledronic acid in patients with prostate or breast cancer [76] and a phase II study of patients with metastatic hormone receptor-negative or locally advanced unresectable breast cancer [77]. Results of these studies will be eagerly anticipated.

### 10.3. Cathepsin K

Cathepsin K is a serine protease, which is highly expressed by activated osteoclasts and is necessary for the degradation of bone matrix proteins [78]. Inhibition of cathepsin K has been shown to inhibit bone resorption in preclinical animal models [79]. Given that cathepsin K is frequently upregulated in breast cancer and is associated with more invasive disease and increased risk of bone metastasis [80,81], it has become a clinical therapeutic target of interest. Use of the cathepsin K inhibitor, odanacatib, was recently evaluated in women with breast cancer and metastatic bone disease. Patients were randomized 2:1 (double-blind) to oral odanacatib 5 mg daily for four weeks or intravenous zoledronic acid 4 mg given once at study initiation [82]. Evaluation of circulating levels of bone turnover markers (urinary *N*-telopeptide) demonstrated that odanacatib was equally effective in suppressing bone turnover markers as zoledronic acid in this short-term study and was well-tolerated. Thus additional analyses testing its efficacy alone or in combination with standardly used bone-targeting agents is warranted.

### 10.4. Transforming Growth Factor-Beta Signaling

The transforming growth factor-beta (TGF-β) family of proteins are known to play a significant role in tumour progression, including the ability to control tumor cell invasion, epithelial to mesenchymal transition and response to therapy [83]. It is also a prime driver of the vicious cycle in bone metastases. A number of inhibitors of TGF-β signalling pathways have been developed and are at various stages of preclinical testing for efficacy in bone metastasis control. Both blockade of TGF-β ligands [84] and inhibition of TGF-β receptor signalling [85,86,87] have demonstrated efficacy in inhibiting bone metastases in models of breast cancer.

### 10.5. Chemokine Receptor Signaling (CXCL-12/CXCR4)

CXCR4 is a transmembrane G protein-coupled receptor, which has gained recent interest in the cancer metastasis world. Studies have shown that tumour cells that express CXCR4 preferentially metastasize to the bone, where its ligand, CXCL12, is abundantly expressed [88]. The subsequent CXCL12/CXCR4 signaling also enhances cell survival via Akt activation, thereby providing an advantage to newly arrived tumor cells in the bone microenvironment [89]. Disruption of the CXCR4 signaling pathway using neutralizing antibodies or synthetic peptide antagonists have been shown to reduce the formation of lung and bone metastases caused by CXCR4-expressing breast cancer cells in preclinical models [90]. Clinically, a phase I/II clinical trial has determined the tolerability and safety profile of repeated administration of a CXCR4 peptide antagonist in a small cohort of patients (*n* = 25) with advanced metastatic disease. Some patients had stable disease and tolerability was good [91]. However, the efficacy of CXCR4 blockade in bone metastatic breast cancer patients will await determination in future clinical studies.

## 11. Optimization of Currently Available Bone-Targeted Therapies

Many questions regarding the optimization of bone-targeted therapy still remain, especially for the use of bisphosphonates in an era of personalized medicine, where the, “one size fits all approach” of 3–4 weekly systemic therapy from diagnosis of bone metastases until death is no longer ideal [92]. Key questions for both physicians [93] and patients [94] that are currently under investigation include questions on optimal timing and dosing of bone-modifying therapy and what to do with this therapy upon documented disease progression.

### 11.1. De-Escalation of Bone-Targeted Agents

Therapy de-escalation in appropriate patients is an attractive option, as it has the potential to improve patient quality of life, reduce drug toxicity and to be more fiscally responsible to individual healthcare systems. This issue was investigated in a phase 3, open label, randomised, non-inferiority trial looking at the efficacy and safety of 12-weekly *versus* 4-weekly zoledronic acid for prolonged treatment of patients with bone metastases from breast cancer (the ZOOM trial) [95]. This trial demonstrated that the skeletal morbidity rate (SMR) was numerically very similar (but statistically non-inferior) in the group of patients who had their zoledronic acid treatment de-escalated to every 12 weeks, as opposed to maintaining it at every four weeks after at least one year of prior treatment, *i.e.*, a less-intensive treatment regimen was equivalent to the standard regimen in women with breast cancer and bone metastases for the primary study endpoint.

A recent systematic review, including the ZOOM data, confirmed that due to the heterogeneity of the current published trials, there is currently insufficient evidence to make wide-spread practice changes to less frequent dosing of bone-targeted agents [96]. Other trials addressing this topic, including TRIUMPH (a multicentre study assessing 12-weekly intravenous bisphosphonate therapy in women with low risk bone metastases from breast cancer using bone resorption markers) [97] and OPTIMIZE-2 (a study of zoledronic acid administered monthly *versus* every 3 months in multiple myeloma and breast cancer patients who were treated with zoledronic acid the prior year) [98], address de-escalation in patients already established on bisphosphonate therapy, while trials, like the Cancer and Leukemia Group B (CALGB) 70604 trial [99], address the de-escalation question in bisphosphonate naive patients.

### 11.2. Switching Strategies

A common clinical question is whether or not to switch bone-targeted agents in patients with either disease progression or occurrence of an SRE, while on a bone-targeted therapy. Data from phase III trials addressing this question is currently lacking. To date, the only data that can answer the question of “switching” are small phase II trials, and definitive conclusions cannot be drawn from them [47,100,101,102]. Switching to a more potent bisphosphonate or from a bisphosphonate to denosumab after progression stands as an attractive alternative; however, such a strategy cannot be definitively recommended or refuted until a large adequately powered clinical trial with appropriate endpoints is completed to test this hypothesis. Our group is currently conducting a randomized, double-blind, placebo-controlled, phase III trial evaluating the palliative benefit of either continuing pamidronate or switching to second-line zoledronic acid in breast cancer patients with high risk bone metastases (the Odyssey Study) [103]. The primary objective of this study is to compare the proportion of high-risk metastatic breast cancer patients with bone metastases that will achieve a decrease in serum *C*-telopeptide (a surrogate for decreased risk of SREs) in the zoledronic and pamidronate treatment arms. Secondary objectives include determining the proportion of high-risk metastatic breast cancer patients that will achieve a significant improvement in palliative response and assessment of the overall pain control between the experimental group and the control group over the 12-week study period.

## 12. Conclusion

Bone metastases in breast cancer are common and are associated with increased morbidity and poor prognosis. Directed therapy at minimizing such complications plays a pivotal role in the management of these patients. Over recent decades, increased understanding of the pathological processes involved in bone metastases behavior has led to effective and safe bone-targeted therapies becoming available for use in the palliative management of patients with breast cancer and bone metastases. Based on clinical trial data, at this time, both bisphosphonates and denosumab are commonly used. They have been shown to reduce the number, and delay the onset, of SREs. Both options are relatively safe, as well, and the choice of which one to pursue should be individualized on a patient to patient basis based on factors, such as; personal choice, availability of agent, cost, route of administration, compliance, co-morbidity and clinician preference. It should be noted, however, that neither the bisphosphonates nor denosumab have been shown to affect either progression-free or overall survival.

Patients with bone only metastases from breast cancer may survive for many years, so long-term toxicities from bone-targeted therapies are important; especially since these are given in the palliative setting. The potential for toxicity should always be considered against the relative benefit of therapy and all efforts made to identify risk factors that predispose to toxicity and managing them appropriately prior to initiation of bone-modifying therapy.

Ongoing studies on the treatment of bone metastases are focused on two areas: Firstly, studies designed to develop a better understanding of the underlying pathophysiology of bone metastasis so that new agents may be developed; and secondly, those utilizing our currently available therapies in a more patient-centred manner. As clinicians in an era where personalized medicine is becoming more widespread, it is time to move away from the thinking of a “one size fits all” approach and start individualizing our treatments to maximize benefit in our patients while simultaneously minimizing unnecessary toxicity. These findings should also hopefully translate into improved care for patients with other malignancies that commonly spread to bone [104]. Studies evaluating novel agents and treatment strategies are ongoing, and the results of these studies are awaited with great anticipation. 
